# Young Folks’ Physiology

**Published:** 1885-03

**Authors:** 


YOUNG FOLKS' PHYSIOLOGY—OUR BODIES; OR, HOW WE LIVE. An Elementary Text-Book of Physiology and Hygiene for use in the Common Schools, with Special Reference to the Effects of Stimulants and Narcotics on the Human System. By Albert F. Blaisdell, M. D. Boston: Lee and Shepard, publishers; New York: Charles T. Dillingham, 1885.

This is a small duodecimo volume, of 276 pages. It is bound in a very attractive and inviting muslin; but the young folks allured by this will be still more charmed and delighted by its contents. The style is plain, pointed and crisp; and the system and arrangement of the topics treated of are admirable. It is profusely and elegantly illustrated. It is intended for the tyro in science, but whosoever is fortunate enough to master everything so well treated of in this book will no longer occupy the position of a beginner, and is then well qualified to appreciate more elaborate treatises upon this most interesting branch of the Natural Sciences. "Our Bodies" is divided into fourteen chapters. The introduction, in a very interesting and simple way, gives the student an insight into the nature, properties and composition of the curiously wrought house which he or she has lived in so long, without once dreaming of its complex, and yet automatic, machinery. The second and third chapters explain the anatomical, as well as physiological, relations of the bones and muscles. The next nine chapters treat of the usual subjects belonging purely to physiology, in a masterly, instructive and striking way. It also contains a chapter of plain and homely hints on every-day matters of health; an entire chapter devoted to a systematic series of practical experiments, with full explanations, hints and helps. There are test questions for review at the end of each chapter, which should prove to be very useful. There are many interesting supplementary notes; also review topics, blackboard exercises, and diagrams; glossary; index; in fact it is what it claims to be: a good, practical, accurate text-book, and will doubtless ere long find its way into our grammar schools in the South, as well as in the North, where it is already being extensively used.

Anatomy, physiology and hygiene are subjects which, treated in this way, can be understood by the smallest school-boy or girl, and ought to be essentials of school education.

